# Rhinomanometry: A Comprehensive Review of Its Applications and Advancements in Rhinology Practice

**DOI:** 10.7759/cureus.61370

**Published:** 2024-05-30

**Authors:** Nimisha Patil, Shraddha Jain

**Affiliations:** 1 Otolaryngology-Head and Neck Surgery, Jawaharlal Nehru Medical College, Datta Meghe Institute of Higher Education and Research, Wardha, IND

**Keywords:** technological advancements in rhinology, nasal obstruction, rhinology diagnostics, nasal resistance, nasal airflow, rhinomanometry

## Abstract

Rhinomanometry is a pivotal diagnostic technique in rhinology, providing a quantitative assessment of nasal airflow and resistance. This review comprehensively examines the historical development, principles and clinical applications of rhinomanometry, emphasising its role in diagnosing nasal obstructions, preoperative evaluations and monitoring therapeutic outcomes. Recent advancements, including the integration with imaging technologies and the application of artificial intelligence (AI), have significantly enhanced the accuracy and utility of rhinomanometry. Despite facing challenges such as technical limitations and the need for standardisation, rhinomanometry remains an invaluable tool in both clinical and research settings. The review also explores future directions, highlighting the potential for device miniaturisation, telemedicine integration, personalised protocols and collaborative research efforts. These advancements will likely expand the accessibility, accuracy and clinical relevance of rhinomanometry, solidifying its importance in the ongoing evolution of rhinology practice.

## Introduction and background

Rhinomanometry is a diagnostic technique used to assess nasal airflow and resistance objectively [1]. It involves measuring the pressure and airflow within the nasal cavity, providing valuable insights into nasal function. By quantifying nasal airflow parameters, such as resistance and volume, rhinomanometry aids in the evaluation of various nasal conditions, including nasal obstruction, allergic rhinitis and septal deviations [2].

In rhinology practice, the assessment of nasal function is crucial for diagnosing and managing a wide range of nasal disorders [3]. Rhinomanometry offers a non-invasive and quantitative method for evaluating nasal patency and identifying abnormalities in airflow dynamics. It provides objective data that can guide treatment decisions, surgical planning and monitoring of therapeutic outcomes. Additionally, rhinomanometry serves as a valuable tool for research purposes, contributing to advancements in understanding nasal physiology and pathophysiology [4].

The purpose of this review is to provide a comprehensive overview of the applications and advancements of rhinomanometry in rhinology practice. By examining the principles, clinical applications and recent developments in rhinomanometry, this review aims to highlight its significance as a diagnostic tool and its evolving role in the management of nasal disorders. Furthermore, it intends to discuss the challenges and future directions in rhinomanometry research and clinical practice, offering insights into potential areas for innovation and improvement.

## Review

Historical background

Early Development of Rhinomanometry

The origins of rhinomanometry trace back to the late 19th and early 20th centuries. In 1882, Paulsen conducted seminal research on nasal airflow, marking the nascent experimental phase of rhinomanometry [5]. The 1920s witnessed a significant milestone with Aschan and Hendolin's introduction of the first electronic rhinomanometer, ushering in an era of more precise measurements of nasal airflow and resistance [6]. This period marked substantial progress in the field, laying the groundwork for the standardised techniques prevalent in modern rhinology practice. Moreover, the historical evolution of rhinomanometry included standardisation efforts initiated by Kern in the late 1970s and early 1980s and further developed by Clement and other researchers globally in subsequent years [6]. These standardisation initiatives aimed to establish consistent and physiologically sound techniques for rhinomanometry, ultimately leading to the widespread adoption of active anterior rhinomanometry as the predominant and dependable method for assessing nasal airflow resistance [6]. The early development of rhinomanometry was characterised by pioneering contributions from researchers such as Paulsen, technological breakthroughs in electronic instrumentation by Aschan and Hendolin and subsequent standardisation endeavours by Kern and Clement. Collectively, these efforts laid the foundation for the contemporary use of rhinomanometry as a standard diagnostic tool for objectively evaluating nasal function [6,5].

Milestones in Rhinomanometry Research

Rhinomanometry boasts a rich historical lineage dating back to the early 20th century, punctuated by pivotal milestones that underscore its evolution. The inaugural rhinomanometric measurements, conducted by Zwaardemaker in 1889 utilising a water manometer, laid the groundwork for objectively assessing nasal airflow. Fast forward to the 1920s, Aschan and Hendolin's pioneering development of the first electronic rhinomanometer heralded a transformative phase marked by technological advancements in nasal airflow measurement. Continued innovation unfolded in subsequent decades, notably with Broms' introduction of active anterior rhinomanometry in 1982, bolstering this diagnostic instrument's precision and clinical utility [7]. Concurrently, the 1970s witnessed the advent of acoustic rhinometry, spearheaded by Jackson et al., revolutionising nasal geometry assessment through the utilisation of reflected sound waves. This technique offered a non-invasive and objective avenue for evaluating nasal patency, complementing traditional rhinomanometric approaches. The 1990s brought forth the emergence of a four-phase rhinomanometry (4PR), facilitating the assessment of nasal resistance across the respiratory cycle and providing a more holistic comprehension of nasal function [8]. Recent years have seen concerted standardisation endeavours by entities such as the European Rhinologic Society, refining and validating protocols for both rhinomanometry and acoustic rhinometry to ensure the consistency and reliability of measurements. These advancements have firmly entrenched rhinomanometry's position in contemporary rhinology practice as an indispensable tool for diagnosing and managing nasal and sinus disorders. Despite its venerable history and numerous breakthroughs, ongoing research and technological innovations persist in expanding the capabilities and applications of rhinomanometry within the realm of otorhinolaryngology [9].

Evolution of Rhinomanometry Techniques

The evolution of rhinomanometry techniques has undergone notable advancements, reflecting a continuous quest for precision and reliability in nasal airflow assessment. Initially dubbed 'passive rhinomanometry', this method, dating back to Kayser's work in 1895, found particular utility in paediatric applications [10]. However, the advent of computer-aided rhinomanometry circa 1980 marked a significant breakthrough in functional diagnostics, supplanting earlier graphical methodologies and ushering in integrating personal computers into daily practice [11]. This transition uncovered technical inaccuracies, notably in repeated loops evident in recorded XY curves, prompting the demand for more sensitive and expeditious digital sensors for pressure and mass flow measurements [11]. In 1994, Vogt and Hoffrichter introduced the concept of 'high-resolution rhinomanometry', which entailed dissecting the breathing cycle into four distinct phases to heighten precision and dependability in measurements [11]. This innovative approach aimed to rectify errors stemming from technical equipment and facilitate a more nuanced analysis of nasal airflow dynamics. Validation through model experiments and computational fluid dynamics (CFD) simulations subsequently affirmed the efficacy and technical robustness of four-phase rhinomanometry (4PR) as a diagnostic modality for probing nasal airflow physiology [11]. The evolution of rhinomanometry culminated in establishing 4PR as a standard methodology adopted in clinical rhinology, plastic surgery and sleep medicine across over 20 countries [11]. The central tenets of 4PR encompass replacing estimations with measurements, introducing parameters pertinent to subjective obstruction sensing and furnishing graphical insights into disturbances in nasal valve function [11]. These advancements have profoundly elevated rhinomanometry's accuracy, reliability and clinical relevance in appraising nasal patency and informing treatment strategies.

Clinical applications

Assessment of Nasal Obstruction

The evaluation of nasal obstruction entails the objective scrutiny of symptoms related to nasal congestion, often employing instruments such as the Nasal Obstruction Symptom Evaluation (NOSE) scale. Widely employed in otorhinolaryngology, this scale is a dependable means of appraising nasal patency in individuals afflicted with nasal disorders, furnishing clinicians with a reliable and validated tool [12]. A study concentrating on surgically assisted rapid maxillary expansion (SARME) applied the NOSE scale to gauge nasal obstruction symptoms among patients undergoing this intervention prospectively. The findings revealed the subjective enhancements or absence of deterioration in nasal obstruction symptoms post SARME, underscoring the efficacy of this therapeutic approach in ameliorating nasal patency [12]. Moreover, the objective evaluation of nasal obstruction is paramount in clinical practice, particularly in diagnosing various nasal maladies such as allergic rhinitis, sinusitis, nasal polyps and structural anomalies such as septal deviation or nasal tumours [7]. Nasal obstruction can precipitate mouth breathing, oral dryness, nasal speech, snoring and potentially obstructive sleep apnoea, underscoring the significance of accurate assessment and intervention for this condition [7]. In primary care settings, the clinical assessment of nasal obstruction entails a comprehensive examination of the nasal cavity. This includes external inspection to detect deformities, anterior rhinoscopy to evaluate mucosal surfaces and turbinates and specific manoeuvres such as Cottle's manoeuvre to pinpoint causes of nasal valve collapse contributing to obstruction [13]. Additionally, further investigations, such as nasal endoscopy, may be warranted in cases where the aetiology of obstruction remains uncertain or neoplastic pathologies are suspected [13]. The assessment of nasal obstruction encompasses both subjective and objective parameters, incorporating tools such as the NOSE scale for symptom appraisal and clinical examinations to identify underlying aetiologies and inform tailored management strategies for individuals grappling with nasal congestion concerns [7,12,13].

Preoperative Evaluation in Rhinoplasty

Preoperative assessment in rhinoplasty is a pivotal phase in securing favourable outcomes for patients undergoing nasal surgery. This assessment commences with a meticulous review of the patient's medical history, encompassing any underlying health conditions or prior nasal surgeries that may influence the surgical approach. A comprehensive grasp of the patient's nasal history is imperative for tailoring the surgical strategy to their specific requirements and ensuring optimal results. Furthermore, a thorough examination of the nasal anatomy from various perspectives is conducted to assess the patient's nasal structure comprehensively. Radiological evaluations, such as CT scans, may scrutinise nasal structures meticulously, particularly in instances involving deviated noses or intricate anatomical concerns [14]. Goal establishment assumes a paramount role in the preoperative evaluation process. Surgeons dialogue with patients to discern their aesthetic aspirations, desires and expectations regarding the surgery. Ensuring these objectives are realistic and harmonise with the patient's facial aesthetics and functional requisites is critical. Preoperative planning entails devising a surgical blueprint based on the patient's objectives and insights from the nasal analysis. This encompasses the selection of appropriate surgical methodologies, materials and operative objectives to attain the desired outcome. Utilising digital imaging techniques can furnish visual representations of prospective surgical outcomes, aiding in decision-making and fostering effective communication with the patient [15]. A final consultation culminates in the review of the surgical plan, ensuring the patient comprehends the procedure's limitations, potential complications and anticipated postoperative trajectory. This consultation serves as an opportunity to address lingering concerns, elucidate expectations and forge a robust patient-surgeon rapport grounded in trust and mutual understanding. By adhering to a comprehensive preoperative assessment regimen, surgeons can optimise surgical outcomes, mitigate risks and augment patient contentment with the results of their rhinoplasty intervention [15].

Monitoring Nasal Airway Function in Allergic Rhinitis

The clinical applications of monitoring nasal airway function in allergic rhinitis entail using objective methodologies such as rhinomanometry to assess nasal patency quantitatively. Rhinomanometry, a technique for measuring nasal airflow and resistance, is critical in diagnosing and surveilling allergic rhinitis, allergy vaccination and pharmacotherapy. Research indicates that nasal resistance escalates with age and correlates with the sensation of nasal obstruction and septum deviation. Additionally, rhinomanometry can discern alterations in nasal patency after allergy vaccination or administering medications such as captopril. Despite its adeptness in delivering sensitive and specific nasal measurements, rhinomanometry is more aptly suited for in-clinic assessments in clinical trials rather than home monitoring [2]. Moreover, acoustic rhinometry, another modality for gauging nasal patency, furnishes nonphysiological metrics of nasal luminal volume and cross-sectional area. While acoustic rhinometry may not be optimal for home monitoring, it boasts advantages in terms of user-friendliness, repeatability and minimal requisite patient cooperation. These objective techniques, encompassing rhinomanometry and acoustic rhinometry, serve as invaluable instruments for appraising nasal obstruction and tracking nasal physiology in allergic rhinitis. They offer insights into the nasal function and contribute to evaluating therapeutic interventions [2,16].

Evaluating Response to Medical Treatment

The evaluation of patient response to medical treatment, particularly concerning endometriosis-associated pain, entails the assessment of diverse outcomes such as pain reduction, recurrence rates, satisfaction with treatment and discontinuation rates due to adverse events or perceived lack of efficacy [17]. Research indicates that a considerable proportion of females with endometriosis derive limited or sporadic benefits from medical therapies, underscoring the necessity for more comprehensive and patient-centred outcome measures to gauge treatment effectiveness for this condition [17]. Furthermore, investigations into understanding the heterogeneity of treatment effects (HTE) across patients have revealed that stratifying risk can facilitate the detection of clinically significant variances in treatment-related benefits and adverse effects. This provides invaluable insights for clinicians and patients in making personalised treatment decisions [18]. By scrutinising data from randomised controlled trials and delineating benefit-harm tradeoffs for patients with varying risk profiles, researchers endeavour to inform clinical decision-making and enhance the customisation of treatments based on individual patient characteristics and anticipated outcomes [18].

Advancements in rhinomanometry

Modern Rhinomanometric Techniques

Modern rhinomanometric techniques represent significant advancements in the field of rhinology. One notable technique is active posterior rhinomanometry with 'head-out' body plethysmography, lauded for its minimally invasive approach to measuring nasal patency [19]. This method involves assessing nasal resistance using a formula (R = 0.78 × (delta P / V)^1.33^), offering valuable insights into nasal obstruction and patency [19]. Additionally, parameters such as nasal airflow acceleration and peak flow index during resting nasal breathing serve as objective indicators for diagnosing symptomatic nasal obstruction, with considerations for nationality and anthropological characteristics aiding in assessing nasal stuffiness severity [19]. Moreover, anterior rhinomanometry, capable of assessing one nostril at a time, is a prevalent and recommended technique for evaluating nasal airway resistance and identifying asymmetries or abnormalities in nasal airflow resistance [20]. This method proves particularly useful in distinguishing between anatomical and mucosal abnormalities, assessing the efficacy of treatments such as nasal steroid sprays and conducting challenge tests with allergens to diagnose airborne allergies [20]. Modern rhinomanometric techniques offer a comprehensive approach to objectively evaluating nasal function, assessing nasal patency and diagnosing nasal obstructions. These advancements provide clinicians in rhinology with invaluable insights into nasal physiology and pathology, aiding in the formulation of tailored treatment plans and improving patient care.

Integration With Imaging Technologies

Integrating artificial intelligence (AI) with imaging technologies has heralded a paradigm shift in medical imaging, precipitating significant advancements in healthcare [21,22]. AI algorithms can analyse medical images and patient data, generating personalised insights tailored to individual anatomical, physiological and pathological variations [22]. This personalised healthcare approach enhances treatment efficacy and mitigates the risk of adverse effects, ultimately culminating in improved patient outcomes and quality of life [22]. Moreover, AI has facilitated the development of image-guided interventions and surgical procedures by amalgamating preoperative imaging data with real-time intraoperative imaging. Through this integration, AI algorithms provide surgeons with augmented visualisation, navigation assistance and decision support, enhancing surgical precision, mitigating procedural risks and enabling minimally invasive techniques, consequently augmenting patient safety and surgical outcomes [22].

In dentistry, digital imaging has emerged as a cornerstone of technological integration, seamlessly interfacing with contemporary dental software for real-time analysis and treatment planning [23]. Digital X-rays and scans can be directly uploaded into treatment planning software, streamlining procedures such as dental implant placement, orthodontic treatments and intricate surgeries [23]. Incorporating digital imaging with computer-aided design and computer-aided manufacturing (CAD/CAM) systems facilitates the expedited design and fabrication of restorations such as crowns or veneers, enhancing patient experience and practice efficiency [23]. Furthermore, digital imaging has paved the way for teledentistry and remote consultations, affording patients access to expert opinions sans the necessity for multiple physical appointments [23]. The amalgamation of AI and other technologies in dental imaging holds promise for predictive analysis, automated image interpretation, personalised treatment planning and seamless integration with teledentistry platforms [23]. Infrared camera integration, exemplified by solutions offered by Sierra-Olympia, furnishes high-performance infrared imaging solutions applicable across diverse domains, including airborne systems [24]. Their state-of-the-art technology streamlines thermal imaging with seamless integration, featuring low size, weight and power (SWaP) characteristics and extended longevity [24].

Artificial Intelligence and Machine Learning Applications

Recent strides in rhinomanometry have witnessed a profound integration of artificial intelligence (AI) and machine learning technologies within rhinology, fundamentally transforming the measurement and analysis of nasal airflow and resistance. Emerging research underscores the extensive application of AI, machine learning and deep learning models in otorhinolaryngology, particularly in image-based analysis and clinical diagnoses and treatments [25,26]. Notwithstanding these advancements, challenges persist, including the imperative for large, meticulously labelled training datasets and the imperative of rigorous validation of AI-based predictions within otorhinolaryngology [25,26]. Adopting ensemble learning models, which prioritise fairness, ethical considerations and privacy, represents a promising development in AI applications within rhinology [27]. Moreover, the integration of AI in rhinology endeavours to refine existing algorithms, augment clinical decision-making and elevate treatment outcomes through personalised approaches and sophisticated diagnostic capabilities [28]. These advancements underscore the potential for AI to surmount technical constraints within rhinology and significantly contribute to the field's evolution and the enhancement of patient care [28].

Emerging Trends in Rhinomanometry Research

Recent advancements in rhinomanometry have ushered in a new era of precision and sophistication in assessing nasal function. One notable technique, four-phase rhinomanometry (4PR), developed by Vogt and Jalowayski, scrutinises the pressure-flow curve across the four respiratory cycle phases. By analysing inspiration, expiration and two transitional phases, 4PR furnishes a more comprehensive understanding of nasal patency compared to conventional rhinomanometry [29]. Computational fluid dynamics (CFD) simulations of nasal airflow are also emerging as complementary tools to traditional rhinomanometry measurements. While CFD simulations often underestimate nasal resistance compared to in vivo rhinomanometry, they offer invaluable insights into nasal function and can inform treatment planning strategies [19].

**Figure 1:**
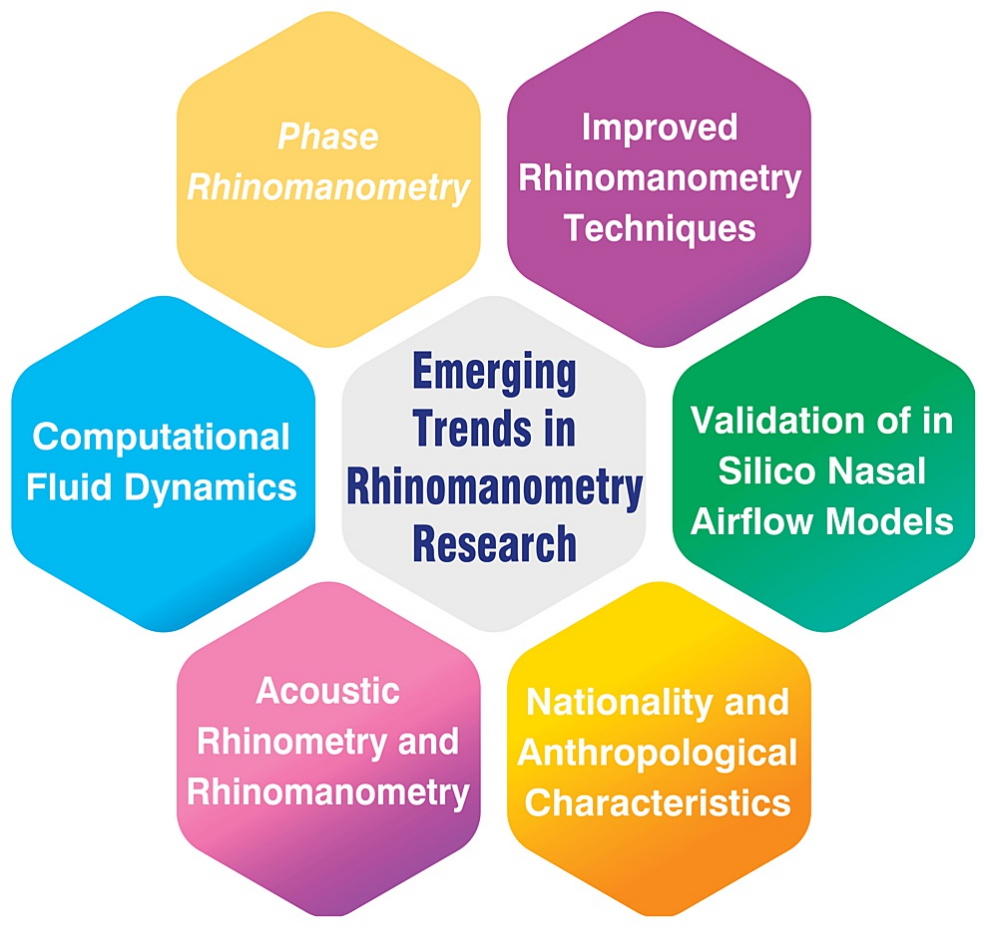
Emerging trends in rhinomanometry research Image credit: Dr. Nimisha Patil

Objective techniques such as acoustic rhinometry and rhinomanometry provide a more reliable assessment of nasal patency than subjective evaluations by patients or clinicians. These techniques serve as guiding beacons in the management of nasal obstruction, aiding clinicians in devising effective treatment regimens [19]. Moreover, studies have shed light on the influence of nationality and anthropological characteristics on the severity and nature of nasal stuffiness, as discerned through rhinomanometry measurements [19]. Furthermore, the validation of in silico nasal airflow models hinges significantly on rhinomanometry. By serving as a benchmark for computational models, rhinomanometry supports research in computational rhinology and facilitates patient-specific treatment planning [19]. Advancements in rhinomanometry techniques continue to refine the diagnostic process. For instance, innovative approaches such as using benzoin tincture to cleanse the nasal skin before testing have streamlined procedure duration and enhanced reliability [19]. These developments underscore the ongoing evolution of rhinomanometry and its pivotal role in shaping the landscape of nasal function assessment and treatment planning. Emerging trends in rhinomanometry research are shown in Figure 1.

Challenges and limitations

Technical Limitations of Current Rhinomanometry Devices

The technical constraints of present-day rhinomanometry devices encompass various facets that affect their functionality and precision. A notable limitation lies in the difficulty of accurately correlating rhinomanometric data with the subjective perception of nasal patency, underscoring the necessity for enhanced calibration and measurement methodologies [30]. Furthermore, the utilisation of active posterior rhinomanometry, while beneficial for evaluating nasal resistance, may encounter its own array of limitations, inaccuracies and measurement challenges, particularly when dealing with intricate nasal conditions such as rhinosinusitis, polyposis processes or septal deviations [29]. Moreover, the disparities between in vivo and in silico measurements in rhinomanometry present a technical hurdle, with computational models often needing to catch up in predicting nasal resistance compared to real-world measurements. This indicates a pressing need for refined computational techniques and modelling approaches [30]. The pragmatic constraints encountered during active anterior rhinomanometry, such as patient discomfort, potential artefacts stemming from mouth breathing and limitations during nasal provocation tests, further compound the technical obstacles encountered by current rhinomanometry devices [31].

Interpretation Challenges

Interpreting rhinomanometry results can present challenges owing to several factors. One notable challenge lies in the disparity between subjective symptoms of nasal obstruction and the objective measurements of nasal patency. While rhinomanometry furnishes valuable insights into nasal resistance and airflow, it may not consistently correlate with a patient's perceived degree of nasal blockage [32]. Additionally, the methodology of active anterior rhinomanometry, which entails assessing nasal resistance during respiration, can encounter practical constraints during testing. These may include interruptions for allergen exposure, artefacts stemming from mouth breathing or issues related to mask fitting [9]. Moreover, interpreting rhinomanometry findings necessitates skilled technicians and optimal patient cooperation, particularly in posterior rhinomanometry. Ensuring controlled ambient conditions, achieving a snug seal of the facial mask and preventing mouth breathing are imperative for attaining reproducible and precise results [33]. Furthermore, identifying abnormalities such as inflammation, nasal polyps or a deviated septum through rhinomanometry demands a careful evaluation by specialists to determine suitable treatments, which may encompass surgical or structural interventions [34].

Patient Factors Affecting Results

Patient-related factors significantly influence the outcomes of nasal airflow assessments and measurements. Various physiological and pathological conditions can impact nasal airflow and resistance, influencing diagnostic tests and evaluations [35]. Variables such as the nasal cycle, septal deviations, turbinate hypertrophy, tumours, synechiae, nasal congestion, allergies, nonallergic rhinitis and sinonasal polyposis can all exert varying degrees of influence on airflow quantity or nasal airway resistance, thus leading to fluctuations in test outcomes [35,36]. Furthermore, individual responses to decongestants offer valuable insights into the underlying causes of nasal obstruction. A lack of response may signify structural obstructions such as septal deviations, bony hypertrophy of the turbinates and inflammatory conditions unresponsive to decongestants [36]. Additionally, the capacity of patients to relax the soft palate during posterior rhinomanometry can significantly impact measurement accuracy, underscoring the criticality of patient cooperation and physiological factors in ensuring the reliability of results [36].

## Conclusions

In conclusion, rhinomanometry is a critical diagnostic tool in rhinology, offering an objective and quantitative assessment of nasal airflow and resistance. This review has highlighted the technique's evolution, clinical applications and recent advancements, emphasising its indispensable role in diagnosing and managing nasal disorders such as nasal obstruction and allergic rhinitis. Integrating modern technologies, such as imaging and artificial intelligence, has significantly enhanced rhinomanometry's accuracy and utility. Despite challenges such as technical limitations and the need for standardisation, rhinomanometry continues to provide valuable insights that guide treatment decisions and surgical planning. Future research and clinical practice will benefit from ongoing innovations, including device miniaturisation, telemedicine integration and personalised protocols. These advancements promise to solidify rhinomanometry's place in rhinology further, ensuring it remains a cornerstone for improving patient outcomes and advancing our understanding of nasal physiology.
